# Raman Spectroscopic and Microscopic Analysis for Monitoring Renal Osteodystrophy Signatures

**DOI:** 10.3390/bios8020038

**Published:** 2018-04-08

**Authors:** John D. Ciubuc, Marian Manciu, Avudaiappan Maran, Michael J. Yaszemski, Emma M. Sundin, Kevin E. Bennet, Felicia S. Manciu

**Affiliations:** 1 Department of Physics, University of Texas at El Paso, El Paso, TX 79968, USA; jdciubuc@miners.utep.edu (J.D.C.); mmanciu@utep.edu (M.M.); emsundin@miners.utep.edu (E.M.S.); 2 Department of Biomedical Engineering, University of Texas at El Paso, El Paso, TX 79968, USA; 3 Border Biomedical Research Center, University of Texas at El Paso, El Paso, TX 79968, USA; 4 Department of Orthopedic Surgery and Biomaterials and Histomorphometry Core Laboratory, Mayo Clinic, Rochester, MN 55905, USA; Maran.Avudai@mayo.edu (A.M.); Yaszemski.Michael@mayo.edu (M.J.Y.); 5 Division of Engineering, Department of Neurologic Surgery, Mayo Clinic, Rochester, MN 55905, USA; Bennet.Kevin@mayo.edu

**Keywords:** Raman spectroscopy, renal osteodystrophy, bone composition, statistical analysis

## Abstract

Defining the pathogenesis of renal osteodystrophy (ROD) and its treatment efficacy are difficult, since many factors potentially affect bone quality. In this study, confocal Raman microscopy and parallel statistical analysis were used to identify differences in bone composition between healthy and ROD bone tissues through direct visualization of three main compositional parametric ratios, namely, calcium content, mineral-to-matrix, and carbonate-to-matrix. Besides the substantially lower values found in ROD specimens for these representative ratios, an obvious accumulation of phenylalanine is Raman spectroscopically observed for the first time in ROD samples and reported here. Thus, elevated phenylalanine could also be considered as an indicator of the disease. Since the image results are based on tens of thousands of spectra per sample, not only are the average ratios statistically significantly different for normal and ROD bone, but the method is clearly powerful in distinguishing between the two types of samples. Furthermore, the statistical outcomes demonstrate that only a relatively small number of spectra need to be recorded in order to classify the samples. This work thus opens the possibility of future development of in vivo Raman sensors for assessment of bone structure, remodeling, and mineralization, where different biomarkers are simultaneously detected with unprecedented accuracy.

## 1. Introduction

Abnormalities in bone composition, such as loss of bone mass and changes in bone mineral density, are known to occur in patients with chronic kidney disease–mineral bone disorder (CKD–MBD), renal osteodystrophy (ROD), and osteoporosis [1,2,3,4,5,6,7,8]. Consequently, an important risk factor for such patients is bone fragility and the associated significant increase in bone fractures [1,2,3,4,5,6,7,8]. CKD–MBD is a systemic mineral metabolic disorder associated with CKD that affects bone metabolism and/or the cardiovascular system [7]. Clinically, the presence of CKD–MBD is determined from abnormal changes in the levels of calcium, phosphorus, parathyroid, and vitamin D metabolism [6,7,8]. Research on ROD, a skeletal disorder component of CKD–MBD, includes studies of bone morphologic changes [7]. The related problems of osteoporosis, another metabolic bone disorder, concern the deterioration of mechanical properties, such as compromised bone strength.

Healthy bone is a natural composite consisting predominantly of collagen, which acts as a structural framework, and bone mineral (biological apatite, mainly hydroxyapatite) plus other noncollagen constituents, such as proteins, lipids, vascular elements, cells, and trace ions (e.g., potassium, carbonate, sodium, magnesium, fluoride, chloride, and citrate) [9]. While the bone mineral provides toughness and rigidity to the bone, the collagen assures its tensile strength and flexibility. From biological and clinical perspectives, all of the known abnormalities in bone turnover, mineralization, volume, linear growth, strength, and in vascular and soft tissue calcifications are evaluated under the common terminology of *bone quality* [1,2,3,4,5,6,7,8]. While changes in mineral metabolism and bone structure can develop early in the course of CKD, they worsen with progressive loss of kidney function; the more severe forms of ROD occur in patients with advanced CKD. Related and similarly problematic bone diseases are hyperparathyroid-mediated high-turnover bone disease or osteitis fibrosa cystica, adynamic bone disease, osteomalacia, and mixed uremic osteodystrophy [5,6,7,8].

Determining the pathogenesis of ROD and its treatment efficacy is difficult, since many factors potentially affect bone quality, and in a complicated manner. Their mechanisms of action are still not completely elucidated, nor is there a clear distinction between the main causes of key bone lesions associated with CKD [8,10]. For example, osteoporosis might be confused with osteomalacia, which is a contributor to ROD. However, detailed investigations have shown that there is a distinct difference between bone characteristics in these two cases, with bone that is porous and brittle in osteoporosis, but soft in osteomalacia. Such variation in bone quality is also associated with the ratio of mineral to organic material, which, in the latter case, is much reduced.

Bone histomorphometry is the technique most employed in assessing bone turnover, mineralization, and volume, and in making in vitro diagnostic discriminations. It is based on analysis of biochemical markers (usually using sample staining) of transiliac bone biopsies [11,12]. The invasive nature of the biopsies, the level of experience necessary for pathologists to recognize and classify the abovementioned abnormalities, and the lack of standardized normal values, make obtaining accurate tests still challenging in many laboratories. Therefore, there is still a need for alternative studies in order to achieve improved assessment of bone properties. An appealing approach is the use of optical spectroscopic techniques. One main advantage of such investigations is the fact that they provide information at the molecular level about all constituents at once and without the need for prior staining—a label-free, simultaneous identification of different biomarkers. Therefore, estimations of important bone compositional parameters, such as mineral-to-matrix ratio, carbonate-to-phosphate ratio, mineral crystallinity, the degree to which carbonate replaces the phosphate ions in the mineral lattice, and the amount of collagen crosslinks, can be and were successfully investigated by optical means as reported in the literature [13,14,15,16,17,18,19]. Such results revealed alteration of the composition of bone tissue, with consequent impaired bone microarchitecture and increased fracture risk in metabolic bone diseases [13,14,15,16,17,18,19]. However, the majority of these studies were based on Fourier transform infrared optical spectroscopy (FTIR) [13,14,15,16]. Only recently have they been extended to Raman spectroscopy [17,18,19]. Overall, the literature on such Raman analysis is still quite scarce.

Raman spectroscopy has considerable potential for the development of future optical-fiber-based sensors with better spatial resolution for in vivo ROD detection. One of its immediate advantages is its direct applicability to in vitro measurements of fresh tissue and, consequently, the elimination of the unwanted, potential interference of embedding matrix material (usually polymethyl methacrylate) that is in standard use for FTIR and histomorphometrical sample preparation. In this study, by employing confocal Raman microscopy, we not only identify significant differences in bone composition between healthy (control) bone tissue and ROD bone tissue, but also achieve direct visualization of the main compositional parametric ratios (i.e., mineral-to-matrix and carbonate-to-amide I ratios) and of important constituents such as phenylalanine and calcium. Furthermore, the statistical analysis presented here is very accurate, since it is performed on hundreds of thousands of accumulated Raman spectra. Thus, by combining experimental findings from Raman spectroscopic imaging of biomarkers with statistical analysis, this work presents new ways of obtaining valuable information about bone quality, with the ultimate goal of improving the means of prediction of pathological fracture risk in ROD.

## 2. Materials and Methods

### 2.1. Sample Preparation

After Institutional Review Board (IRB) approval, 10 specimens representing 5 ROD and 5 normal adult human iliac crest bone biopsies were selected from the database at the Mayo Clinic, in Rochester, Minnesota, and embedded, following standard procedures, in polymethyl methacrylate (PMMA). The specimens were from patients within the age range of 67 ± 8 and had been acquired following the rules of the Declaration of Helsinki of 1975 (https://www.wma.net/what-we-do/medical-ethics/declaration-of-helsinki/), revised in 2008. Biopsy specimens had been selected upon evidence of osteomalacia due to ROD (based on their previous histomorphometry evaluations), while postmenopausal healthy women served as the normal group. For spectroscopic analysis, three sections (5 microns thick) were cut using a Leica RM 2265 microtome (Leica Biosystems Inc., Buffalo Grove, IL, USA) from each of the PMMA-embedded sections of the specimens.

### 2.2. Measurements and Equipment

The Raman measurements were acquired with an *alpha 300R WITec* confocal Raman system (WITec GmbH, Ulm, Germany). To avoid sample damage during data acquisition, the 532 nm excitation of a frequency-doubled neodymium-doped yttrium-aluminum-garnet (Nd:YAG) laser was kept at a low power output of about 5 mW. Because of sample roughness, a 20X objective lens (Olympus, Tokyo, Japan) with a numerical aperture of 0.4 was used. The Raman signal was detected by a 1024 × 127 pixel, Peltier-cooled, back-illuminated, and VIS-AR-coated Marconi 40-11 charge-coupled device (CCD) with a spectral resolution of 4 wavenumbers. The *WITec Control 1.60* software, which also controls the piezoelectric stage for sample scanning, was used for the acquisition of confocal mapping data. Arrays of 150 × 150 Raman spectra were recorded for all Raman images using an integration time of 50 ms per spectrum. The Raman mapping images were acquired with 80 µm × 80 µm scan sizes. Specific regions of interest were carefully selected for such measurements, to avoid PMMA interference and to ensure that the trabecular bone was measured.

### 2.3. Computational Analysis

The output data were further processed by using the *WITec Project Plus* package software, which automatically facilitates background subtraction for hundreds of thousands of spectra. For consistency between measurements and elimination of slight differences in laser power used for measurements of different samples, as well as of potential errors introduced by very slight fluctuations in laser power during the rapid confocal Raman acquisitions of so many spectra, normalization was performed with respect to the intensity of the laser line. Isolation of potential outliers (less than 9 spectra out of 22,500 spectra per sample measurement), that usually originate from locally high sample fluorescence, was also done before calculating the biomarker ratios of interest. An in-house algorithm developed in C++ and MATLAB^®^ version r2016a was employed in performing such ratios.

## 3. Results and Discussion

**Figure 1:**
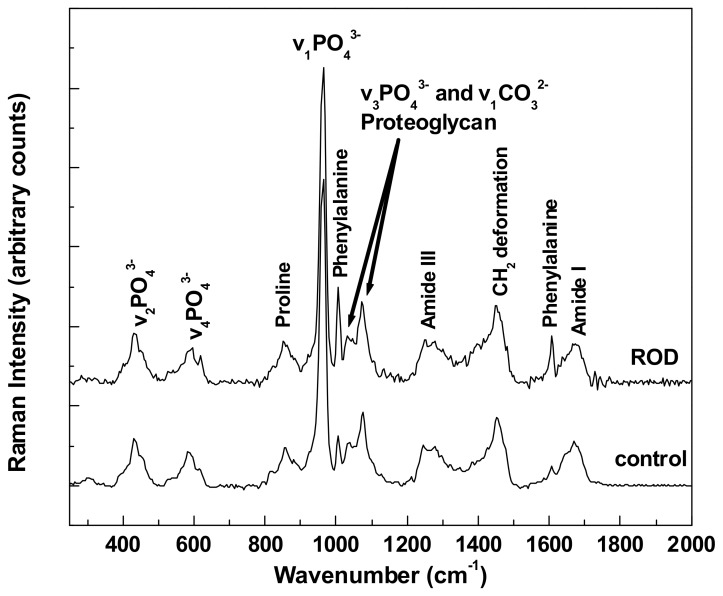
Representative Raman spectra of control and renal osteodystrophy (ROD) bone samples obtained by averaging hundreds of thousands of accumulated spectra.

The overall comparison of differences between all bioconstituents present in healthy (control) bone tissue and ROD bone specimens, which is shown in Figure 1, reveals an obvious distinction regarding the intensities of phenylalanine vibrational lines at 1005 and 1609 cm^−1^. It is known that for patients with a malfunctioning kidney (and/or liver), an increased amount of phenylalanine can be anticipated along with a decrease in tyrosine [20]. This is a consequence of the fact that the phenylalanine hydroxylase (PAH) enzyme, which is located in both kidney and liver and converts phenylalanine into tyrosine via hydroxylation of the aromatic side chain, fails to be expressed properly; thus, the observed excess of phenylalanine in Figure 1 for patients diagnosed with ROD. Reaction with PAH is also the primary means by which the body forms tyrosine from ingested phenylalanine, the latter being an essential amino acid due to the body’s inability to produce it.

The Raman spectra presented in Figure 1 were obtained by averaging multiple integrated spectra of images that were acquired at different locations on the corresponding types of samples, with each integrated Raman image spectrum being the average of 22,500 spectra per image. Thus, these two spectra are outcomes of the averaging of hundreds of thousands of accumulated spectra. Vertical translation and appropriate labeling was applied for easier visualization. Although the raster-scanning employed in acquiring the Raman mapping images for this level of data resolution requires acquisition times of minutes, the results show unprecedented accuracy for detecting potential morphological differences between the ROD and the control specimens. Supporting evidence is the obvious accumulation of phenylalanine in ROD samples reported here, which, to the best of our knowledge, is the first time that this has been observed with Raman spectroscopy. The presence of phosphate, carbonate, proline, and amide bands, which are expected components of the bone structural framework, have also been detected and appropriately labeled. However, no noticeable differences in the respective intensities of these bands are seen for the two types of bone specimens analyzed.

**Figure 2:**
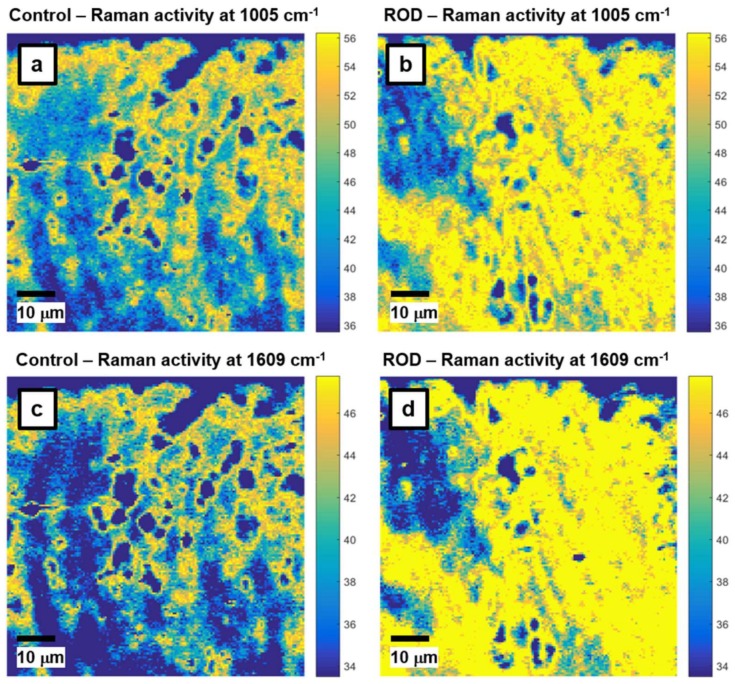
Raman mapping images of phenylalanine activity: (**a**,**b**) with the 1005 cm^−1^ vibrational line, and (**c**,**d**) with the 1609 cm^−1^ band for control and ROD bone samples, respectively. A bright yellow pseudo-color corresponds to a higher intensity.

Since phenylalanine can be a potential ROD signature and a biomarker for identification of the disease, we further analyze its signal in Figure 2a–d, where the locations of 1005 cm^−1^ and 1609 cm^−1^ vibrations are visually presented through associated confocal Raman images for control (Figure 2a,c) and ROD (Figure 2b,d) bone samples, respectively. The bright yellow pseudo-color in these images represents the highest Raman activity associated with the corresponding vibrations, while dark blue corresponds to the lowest Raman activity. Background subtraction and normalization of these images was performed as evidenced by the scaling of the color bars linked to these images. Only the most representative images are presented here. Criteria for this selection were based on patient age and clinical diagnosis as well as prior medical treatment. Besides expected similarities in the images of Figure 2a,c and those of Figure 2b,d, much more extensive yellow regions are observed in the images associated with the ROD specimens, corroborating the corresponding higher Raman activity (i.e., intensity) of phenylalanine Raman vibrations seen in Figure 1 for the ROD samples.

It is also known that for ROD patients a combination of high phosphate levels and low calcium levels, again both attributed to damaged kidney functions, are usually clinically detected. The first are a consequence of the fact that a damaged kidney is unable to excrete phosphate properly, resulting in its systemic accumulation. Furthermore, a damaged kidney is unable to properly convert vitamin D obtained from food (vitamin D3) into its active state of calcitriol, of which the primary function is to increase calcium levels by improving the uptake of calcium in the gut from food and to stimulate kidney reabsorption of calcium. Consequently, the deficit of calcium in blood test analyses of such patients is mainly attributed to kidney malfunction. Since the other potential source of calcium to which the body has access is the bones, lower calcium levels are identified in ROD patients, as the body keeps leeching calcium from the bones and is unable to replenish it at the same rate from external sources (i.e., diet) due to the lack of calcitriol.

**Figure 3:**
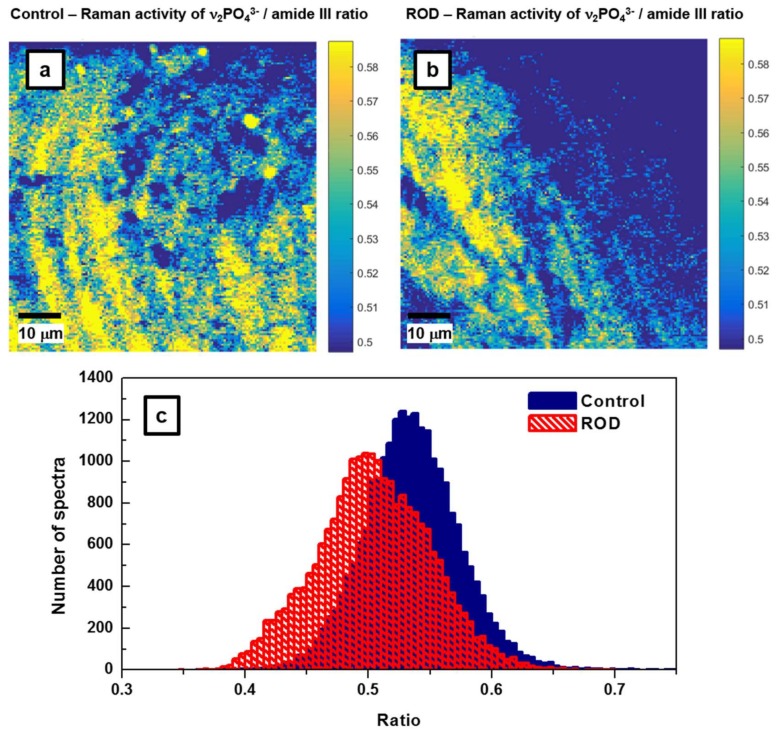
(**a**,**b**) Raman mapping images for the control and ROD bone samples of υ_2_PO_4_^3−^/amide III ratios, which correspond to activities of the bands centered around 430 cm^−1^ and 1275 cm^−1^, respectively. The υ_2_PO_4_^3−^/amide III ratio is associated with calcium content in the samples [21]. (**c**) Statistical analysis associated with Raman mapping images in Figure 3a,b.

With this remark in mind, we analyze in Figure 3a,b the calcium levels in normal and ROD bone samples, respectively. The υ_2_PO_4_^3−^/amide III Raman activity ratio, which corresponds to the ratio of the area under the Raman band around 430 cm^−1^ (from 395 cm^−1^ to 469 cm^−1^) to that under the peak around 1275 cm^−1^ (from 1215 cm^−1^ to 1332 cm^−1^) from Figure 1, was used for such analysis. Roschger et al. reported that this ratio is proportional to the calcium content [21]. Indeed, based on the overall presence of yellow, green, and light-blue regions, a much lower υ_2_PO_4_^3−^/amide III Raman activity ratio is observed in the image corresponding to ROD bone (Figure 3b) than in that of normal bone (Figure 3a). In addition to visually confirming a lower calcium content in the ROD sample, for a more accurate quantification of this assessment, we also present in Figure 3c the associated statistical analysis.

The probability *p* that the ratios of these Raman features on both samples (control and ROD) have the same average values *μ* is calculated via the unpaired *t*-test without assuming that the populations have equal variances (Welch’s *t*-test, subroutine *t*-test 2 in MATLAB^®^ version r2016a) [22]. For the images presented in Figure 3a,b, the corresponding histograms of these ratio values (Figure 3c), assuming that they are normal distributions, lead to *μ_a_* = 0.5358, *σ_a_* = 0.0474, *μ_b_* = 0.5042, *σ_b_* = 0.0456, and the difference of the averages Δ = *μ_a_* − *μ_b_* = 0.0316 with the 95% confidence interval 0.0308 < Δ < 0.0325. The probability *p* that they are sampled from the same distribution (the average ratios are the same in both samples) is negligibly small, *p* < 10^−300^.

The number of spectra per sample needed to have a detection power *β* at a level *α* of statistical significance is [22]:

(1) $$n_{a}=n_{b}=\frac{\left(\sigma_{a}^{2}+\sigma_{b}^{2}\right){\left(Z_{1-\frac{\alpha }{2}}+Z_{1-\beta }\right)}^{2}}{\Delta^{2}}$$

where *Z* is the standard normal distribution, σ_a_ and σ_b_ are the standard deviations for the two samples, and Δ = *μ_a_* − *μ_b_* is the difference between the averages of the samples. Post-hoc power analysis shows that, for a typical detection power of *β* = 90% at the level *α* = 0.05, only about *n* = 46 recorded spectra per sample is sufficient to distinguish between samples.

**Figure 4:**
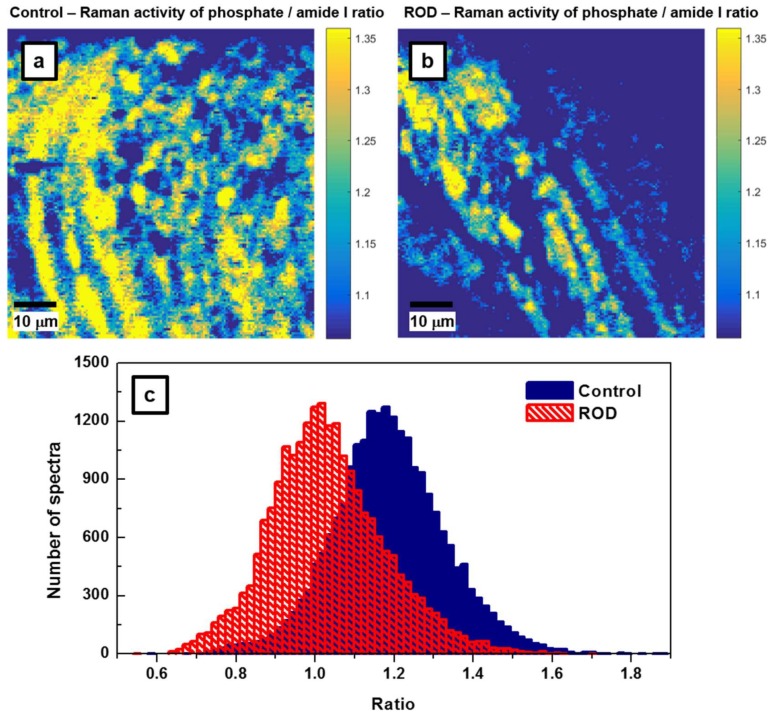
(**a**,**b**) Raman mapping images for the control and ROD bone samples of phosphate/amide I ratios, which correspond to activities of the bands centered around 960 cm^−1^ and 1660 cm^−1^, respectively. This ratio is associated with the mineral-to-matrix content in the samples. (**c**) Statistical analysis associated with Raman mapping images in Figure 4a,b.

Another frequently reported measure of bone quality is the relative mineral to organic content, namely, the mineral-to-matrix ratio [17,18,19]. This ratio, which is based on the integrated area under the phosphate peak at 960 cm^−1^ (from 907 cm^−1^ to 990 cm^−1^) and that of the amide I band around 1660 cm^−1^ (from 1625 cm^−1^ to 1725 cm^−1^), represents the amount of mineral per amount of collagen per volume analyzed. While in the literature previously, the assessment of this ratio was achieved by spectral analysis alone, we extend it here to direct visualization through the associated Raman imaging presented in Figure 4a,b. Since in confocal Raman microscopy the band intensities of some constituents, such as phosphate and collagen amide I bands, are polarization-sensitive [23,24,25], unpolarized recording using a low numerical aperture (NA) objective (i.e., NA = 0.4 of 20X objective) avoids the influence of this effect in addition to that of sample roughness. Furthermore, to minimize the calculation errors, we consider the ratio of areas under the corresponding peaks instead of the ratio of their intensities [17,18,19,21,23,24].

The results presented in Figure 4a,b demonstrate that bone from patients with ROD exhibited significantly smaller amounts of high mineral-to-matrix ratio than did the normal group (i.e., fewer intense yellow regions are depicted in Figure 4b of the ROD image). This behavior is expected in ROD patients with hyperparathyroidism, which results in increased bone resorption and turnover, as well as with hyperosteoidosis. The corresponding histograms of these ratios that are shown in Figure 4c have *μ_a_* = 1.1876, *σ_a_* = 0.143, *μ_b_* = 1.0316, *σ_b_* = 0.145, and the difference of the averages Δ = *μ_a_* − *μ_b_* = 0.156 with the 95% confidence interval 0.1534 < Δ < 0.1587. Again, the *p* value is less than 10^−300^. Post-hoc power analysis (Equation (1)) shows that for *β* = 90% and *α* = 0.05, only about *n* = 18 recorded spectra per sample is sufficient to distinguish between samples. Furthermore, the ROD histogram has a non-Gaussian shape, with a distribution skewed to the lower side of the mean value. This feature, which was previously observed and reported in the literature, has been attributed to a decrease in bone heterogeneity and mineral properties with age [17].

**Figure 5:**
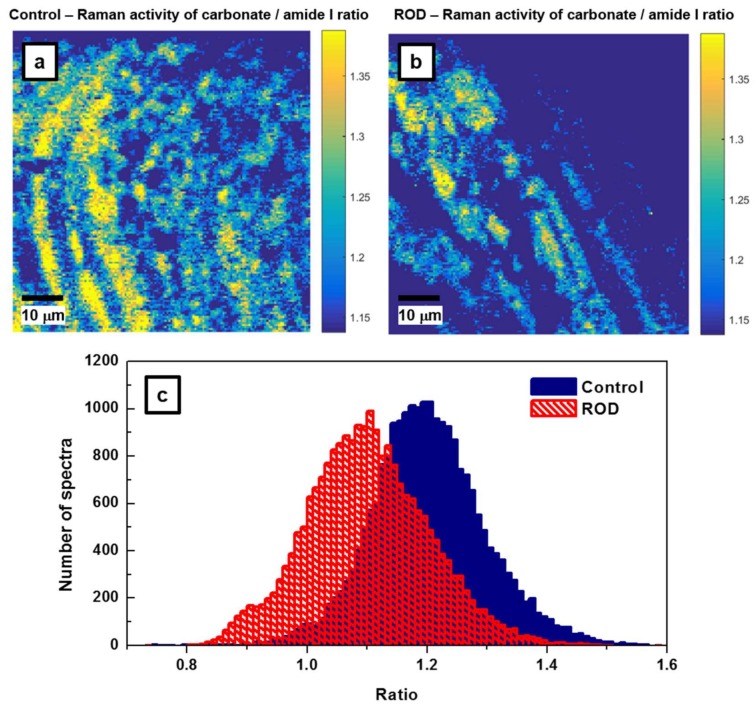
(**a**,**b**) Raman mapping images for the control and ROD bone samples of carbonate/amide I ratios, which correspond to activities of the bands centered around 1074 cm^−1^ and 1660 cm^−1^, respectively. This ratio is associated with bone remodeling and turnover rate. (**c**) Statistical analysis associated with Raman mapping images in Figure 5a,b.

Since the carbonate-to-matrix ratio is also a good indicator of bone turnover and remodeling activity, as well as bone cell counts and osteoid production, we present the confocal Raman images of this biomarker in Figure 5a,b. We were also looking for a potential correlation between the carbonate-to-matrix ratio and the mineral-to-matrix ratio, both being standard parameters usually used for ROD validation. A smaller amount of high CO_3_^2−^/amide I ratio is noticed in the ROD image, consistent with a higher turnover remodeling in ROD samples, and supporting the characteristics of the image in Figure 4b. Areas under the carbonate band around 1074 cm^−1^ (from 1033 cm^−1^ to 1135 cm^−1^) and under the amide I band around 1660 cm^−1^ (from 1625 cm^−1^ to 1725 cm^−1^) were considered for these carbonate to amide I ratios. For further assessment of our findings, the associated statistical analysis of these ratios is presented in Figure 5c. These histograms have *μ_a_* = 1.2023, *σ_a_* = 0.0952, *μ_b_* = 1.1007, *σ_b_* = 0.1016, and the difference of the averages Δ = *μ_a_* − *μ_b_* = 0.1015 with the 95% confidence interval 0.0997 < Δ < 0.1033 (*p* < 10^−300^). The number of spectra per sample required to achieve the typical statistical power (i.e., *β* = 90%, *α* = 0.05) is only about *n* = 20 in this case; this is very similar to that needed for the mineral-to-matrix ratio.

Investigation of the bone collagen matrix in the amide I region in Raman spectroscopy is less precise than in FTIR measurements, with no clear distinction between the 1660 cm^−1^ and 1690 cm^−1^ bands that are used to measure the nonreducible to reducible collagen crosslink ratio [15,17,23,25]. Although we are still attempting to assess the collagen quality parameter by using the band centered around 1660 cm^−1^, no visible difference in collagen dominance between the control and ROD samples was observed (data not presented here). The statistical results also indicate the necessity of a much larger number of spectra per sample (into the hundreds) for a typical statistical power of *β* = 90% and *α* = 0.05. Again, as with the mineral-to-matrix ratio, the histograms associated with the collagen matrix were non-Gaussian, especially that of the ROD sample. By analogy, we also associate this behavior with a decrease of collagen content with age.

The computationally estimated values of the ratios of previously analyzed biomarkers along with the number of spectra per sample required to achieve a statistical detection power of *β* = 90%, at a level of statistical significance *α* = 0.05, are summarized in Table 1 below.

The mineral-to-matrix and the carbonate-to-matrix ratios are the strongest predictors of bone mechanical properties. Therefore, the results obtained here are of clinical relevance, as accurately distinguishing low-turnover (adynamic) bone from high-turnover bone disease is of importance and not easily done with noninvasive modalities [26]. Prevention of fractures with medications may then more effectively be considered, especially for patients with prevalent fractures [7]. Although there are no set published standards, antiresorptive therapy would be an option in high-turnover disease, anabolic treatment with teriparatide in low-turnover disease, and vitamin D uptake in osteomalacia with high turnover [7]. The confocal Raman microscopy of 150 × 150 spectra recorded per sample and the consequent mapping of the calcium content (Figure 3a,b), the mineral to organic content (Figure 4a,b), and the carbonate-to-matrix ratio (Figure 5a,b) presented in this work allow for clear distinction between normal and ROD bone. Even more important, since a relatively low number of spectra per sample is required for the usual statistical power to distinguish between normal and ROD bone (about 20 for these two cases), this raises the possibility of future development and use of in vivo Raman microsensors to determine the status of the bone. The correlation with the results of the “gold standard” method currently used for ROD assessment, histomorphometry, which might be of interest to the medical community, is not presented here, as it would be unlikely that one could acquire both types of measurements at the same location. More significantly, the advantage of multiplexing by using a label-free optical method for such investigations would be lost, in addition to the fact that the labeling required in histomorphometry would definitely influence the S/N of Raman measurements.

## 4. Conclusions

This study contributes to the quest of finding alternative methods of detecting ROD signatures. Through confocal Raman microscopy and statistical analysis, differences in bone composition between healthy (control) and ROD bone tissues were identified with great accuracy. Furthermore, direct visualization of the main compositional parametric ratios of calcium content, mineral-to-matrix, and carbonate-to-matrix were presented and discussed here. Substantially lower values were found in ROD specimens for these representative ratios. In addition, an obvious accumulation of phenylalanine is Raman spectroscopically observed for the first time in ROD samples and reported here. This observation implies that phenylalanine could also be considered as a biomarker for disease monitoring. Since our images are based on 22,500 spectra per sample, not only are the average ratios statistically significantly different for normal and ROD bone, but our method is clearly powerful in distinguishing between the two types of bone samples. If a transition to in vivo Raman measurements is envisioned, the relatively small number of spectra needed in order to classify a sample as “normal” or “ROD”, support the possibility of future optical-fiber-based Raman sensor development. While development of sensors as such is beyond the scope of this article, in the future, we plan to perform a double-blind analysis on bone samples by independently using our algorithm and Raman method and the “gold standard”, histomorphometry, to investigate how the results derived from both approaches compare. To get a step closer to potential in vivo implementation of our method and statistical algorithm, fast acquisition of random Raman spectra of the order of 10^2^–10^3^ in a line depth profile mode are planned for assessing in how many instances the ROD and normal samples would be found statistically significantly different (at *p* = 0.05 level).

Thus, the spectroscopic and statistical analysis presented in this work, the former having the important advantage of simultaneously obtaining valuable information about different biomarkers, creates the needed label-free methodological foundation for the assessment of bone quality. Consequently, it may also open new avenues in providing additional guidance on potential treatment options, with a less invasive, faster, and more precise method of investigation.

## Figures and Tables

**Table 1:** Computationally determined values of the ratios of biomarkers of interest and the number of spectra sufficient to distinguish between samples for typical statistics power of *β* = 90% and *α* = 0.05.

Ratio	Normal		ROD		Δ = *μ_a_* − *μ_b_*	*n*
	Average Value *µ_a_*	Standard Deviation *σ_a_*	Average Value *µ_b_*	Standard Deviation *σ_b_*		
υ_2_PO_4_^3−^/amide III (associated with calcium content)	0.5358	0.0474	0.5042	0.0456	0.0316	46
υ_2_PO_4_^3−^/amide I (mineral-to-matrix)	1.1876	0.143	1.0316	0.145	0.156	18
υCO_3_^2−^/amide I (carbonate-to-matrix)	1.2023	0.0952	1.1007	0.1016	0.1015	20
